# Supplementary material for: Nrf1 is not a direct target gene of SREBP1, albeit both are integrated into the rapamycin-responsive regulatory network in human hepatoma cells
Source: PLoS One. 2023 Nov 27;18(11):e0294508. doi: 10.1371/journal.pone.0294508 (PMC10681226; doi:10.1371/journal.pone.0294508)

Fig 2A-a1-SREBP1:

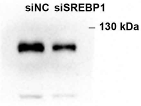

Fig 2A-a2-Nrf1α/β:

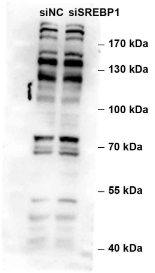

Fig 2A-a3-β-actin:

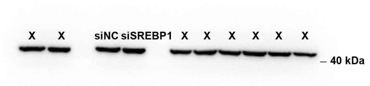

Fig 2B-b1-pS6K1:

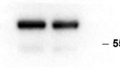

Fig 2B-b2-PSMB5:

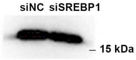

Fig 2B-b3-PSMB6:

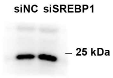

Fig 2B-b4-PSMB7:

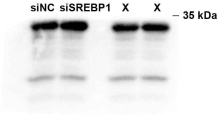

Fig 2B-b5-β-actin:

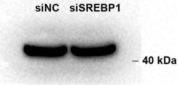

Fig 2C-c1-DDI-1:

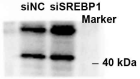

Fig 2C-c2-DDI-2:

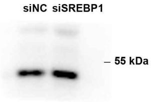

Fig 2C-c3-Hrd1:

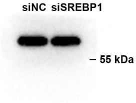

Fig 2C-c4-p97:

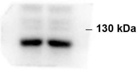

Fig 2C-c5-β-actin:

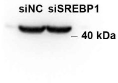

Fig 3B-b1-Nrf1α/β:

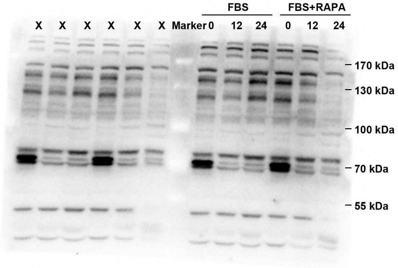

Fig 3B-b2-PSMB5:

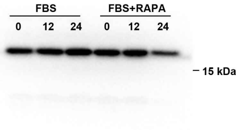

Fig 3B-b3-PSMB6:

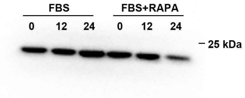

Fig 3B-b4-PSMB7:

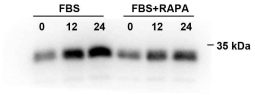

Fig 3B-b5-β-actin:

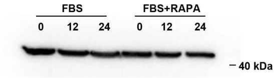

Fig 3C-c1-SREBP1:

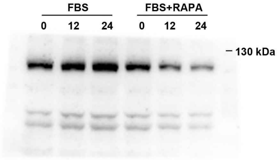

Fig 3C-c2-pS6K1:

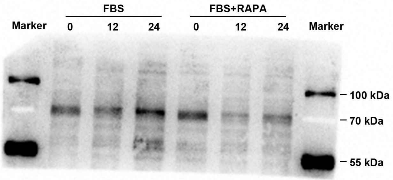

Fig 3C-c3-β-actin:

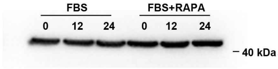

Fig 3E-e1-DDI-1:

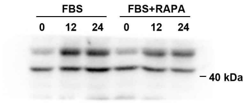

Fig 3E-e2-DDI-2:

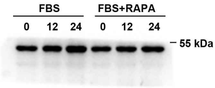

Fig 3E-e3-p97:

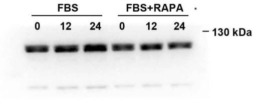

Fig 3E-e4-Hrd1:

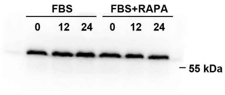

Fig 3E-e5-β-actin:

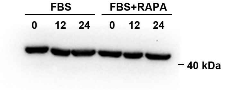

Fig 4C-DDI-1:

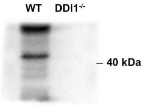

Fig 4C-GAPDH:

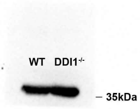

Fig 4G-Nrf1:

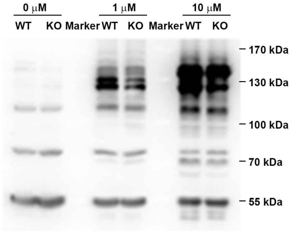

0 μM
1 μM
10 μM
WT
KO
Marker
WT
KO
Marker
WT
KO
170 kDa
130 kDa
100 kDa
70 kDa
55 kDa

Fig 4G-β-actin:

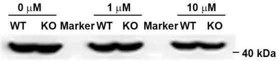

Fig 4H-h1-DDI-1:

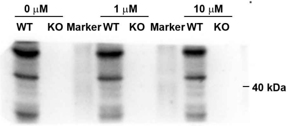

Fig 4H-h2-DDI-2:

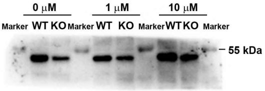

Fig 4H-h3-β-actin:

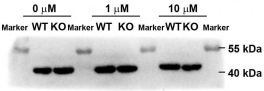

Fig 4H-h4-DDI-1:

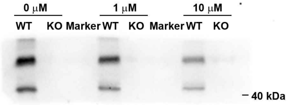

Fig 4H-h5-DDI-2:

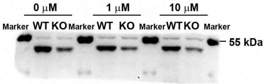

Fig 4H-h6-β-actin:

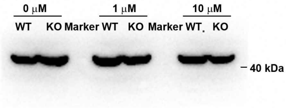

Fig 5A-a1-Nrf1:

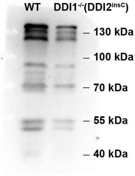

Fig 5A-a2-β-actin:

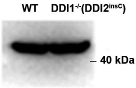

Fig 5A-a3-Nrf1-V5:

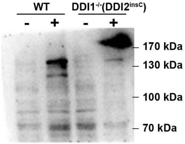

Fig 5A-a4-β-actin:

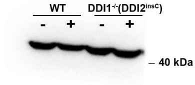

Fig 5A-a5-PSMB5:

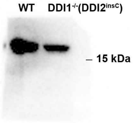

Fig 5A-a6-PSMB6:

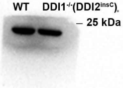

Fig 5A-a7-PSMB7:

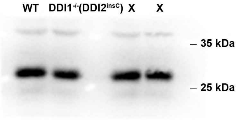

Fig 5A-a8-β-actin:

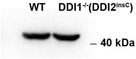

Fig 5B-b1-DDI-2:

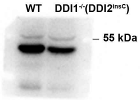

Fig 5B-b2-p97:

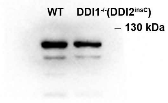

Fig 5B-b3-β-actin:

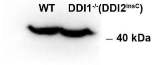

Fig 5B-b4-Nrf2:

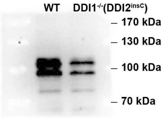

Fig 5B-b5-Keap1:

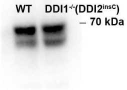

Fig 5B-b6-β-actin:

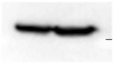

Fig 5D-d1-Nrf1:

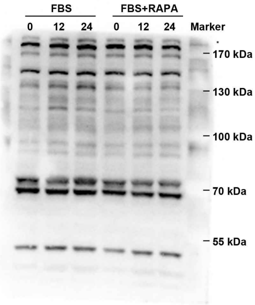

Fig 5D-d2-Nrf2:

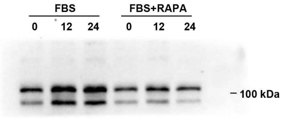

Fig 5D-d3-β-actin:

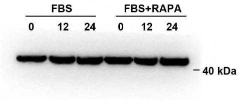

Fig 5E-e1-DDI-1:

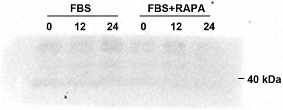

Fig 5E-e2-DDI-2:

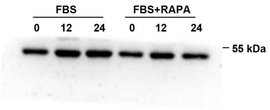

Fig 5E-e3-PSMB5:

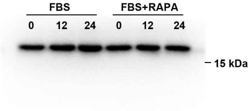

Fig 5E-e4-PSMB6:

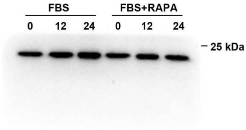

Fig 5E-e5-PSMB7:

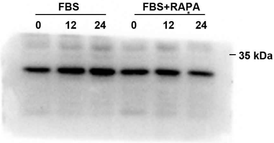

Fig 5E-e6-β-actin:

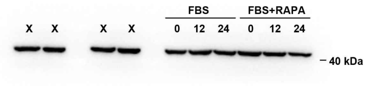

Supplement: S1 File — (PDF) [file pone.0294508.s001.pdf]
