# Supplementary material for: Nrf1 is not a direct target gene of SREBP1, albeit both are integrated into the rapamycin-responsive regulatory network in human hepatoma cells
Source: PLoS One. 2023 Nov 27;18(11):e0294508. doi: 10.1371/journal.pone.0294508 (PMC10681226; doi:10.1371/journal.pone.0294508)
Supplement: S2 File — (PDF) [file pone.0294508.s002.pdf]

Nrf1 is not a direct target gene of SREBP1, albeit both are integrated into the rapamycin-responsive regulatory network in human hepatoma cells

Keli Liu[1,2,3], Shaofan Hu[1,2,3], Lu Qiu[1,3,4], Meng Wang[1,3], Zhengwen Zhang[5], Guiyin Sun[2] and Yiguo Zhang[2,3]*

[1]Bioengineering College, Chongqing University, No. 174 Shazheng Street, Shapingba District, Chongqing 400044, China
[2]Chongqing University Jiangjin Hospital, School of Medicine, Chongqing University, No. 725 Jiangzhou Avenue, Dingshan Street, Jiangjin District, Chongqing 402260, China
[3]The Laboratory of Cell Biochemistry and Topogenetic Regulation, College of Bioengineering, Chongqing University, No. 174 Shazheng Street, Shapingba District, Chongqing 400044, China
[4]School of Life Sciences, Zhengzhou University, No. 100 Kexue Avenue, Zhengzhou 450001, Henan, China
[5]Laboratory of Neuroscience, Institute of Cognitive Neuroscience and School of Pharmacy, University College London, 29-39 Brunswick Square, London WC1N 1AX, England, United Kingdom
* Correspondence to YZ: yiguozhang@cqu.edu.cn

S1 Fig

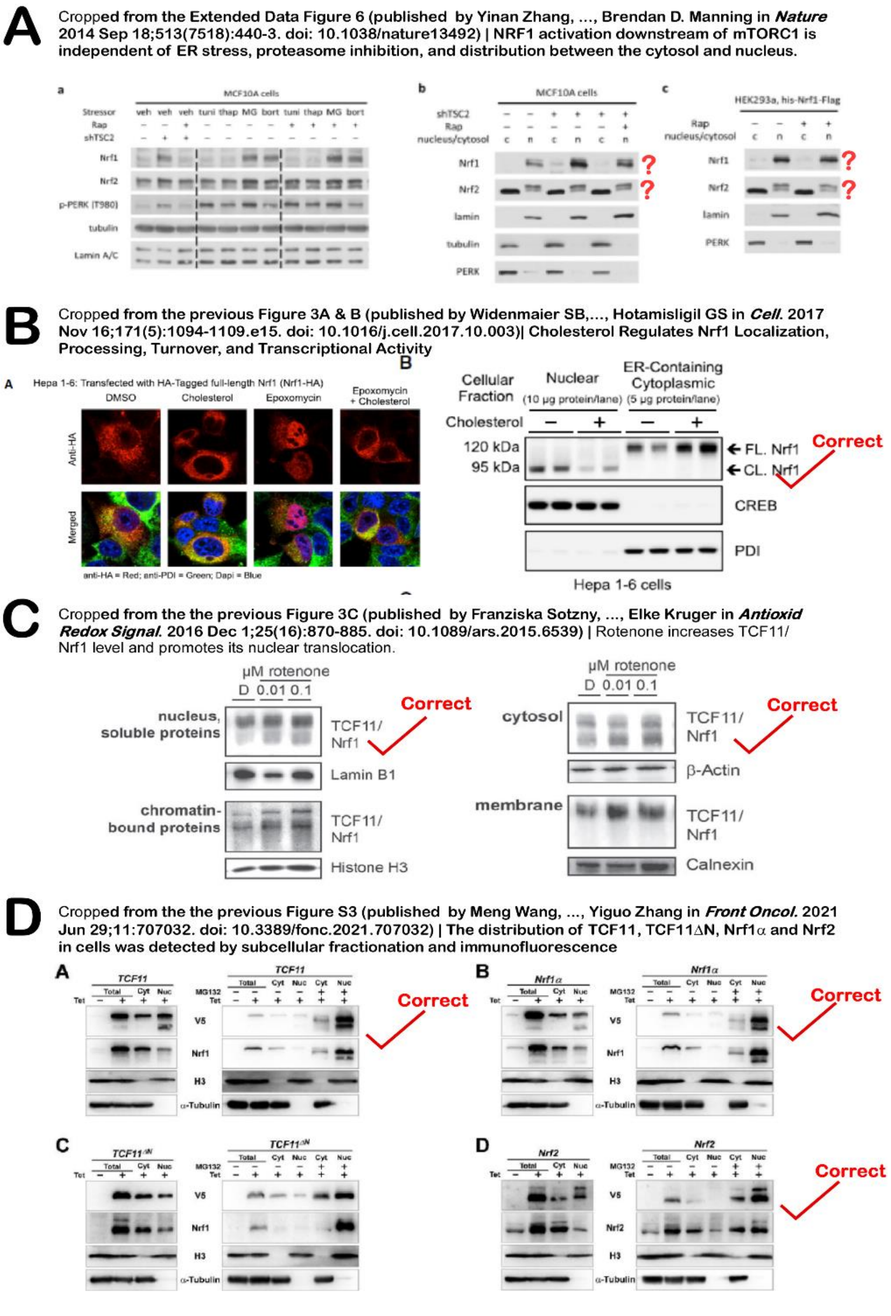

S1 Fig. All the images are cropped from the original publications as indicated here. The relevant contents were also described in the main texts of this paper.

S2 Fig

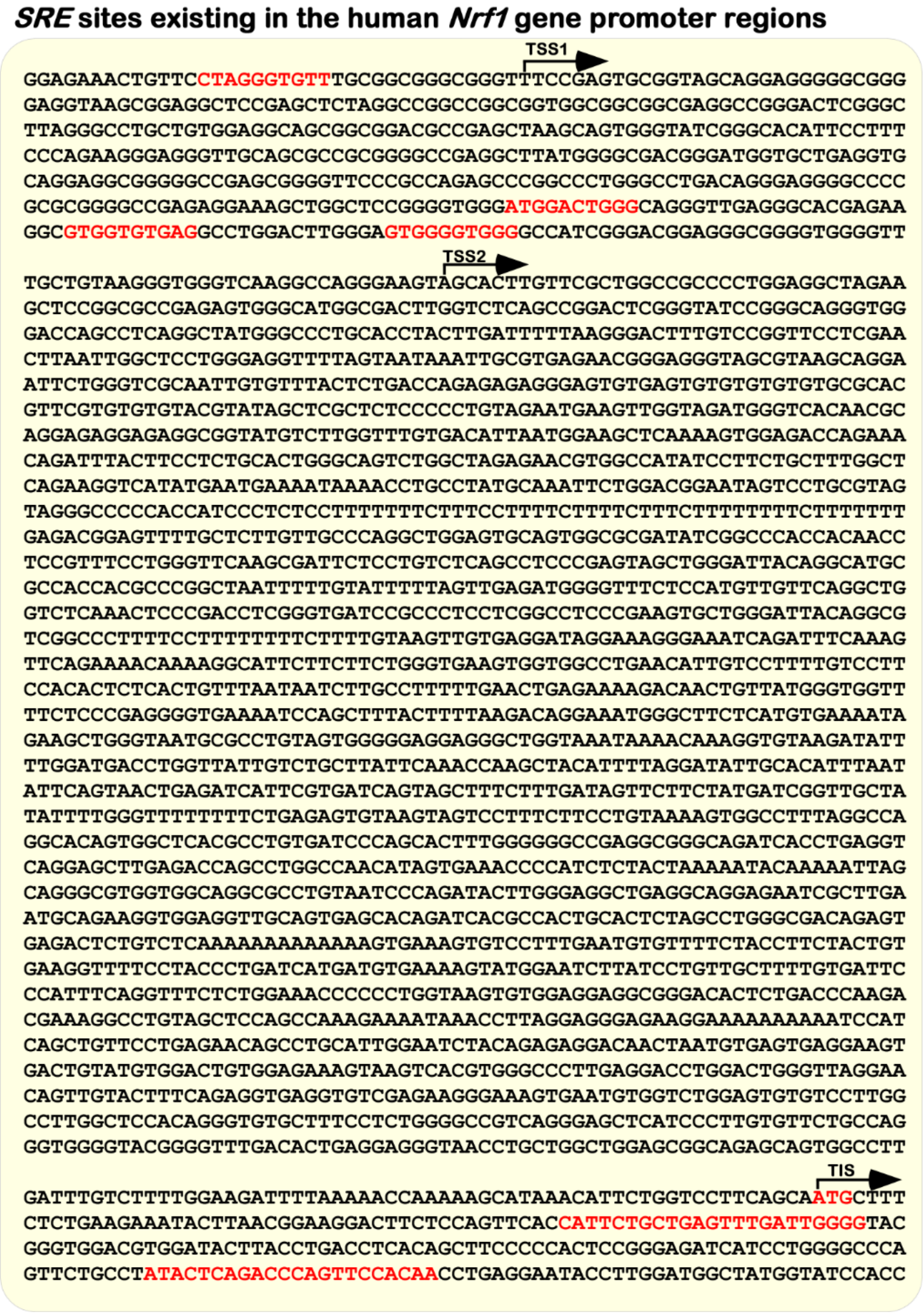

S2 Fig. Several sterol regulatory element (SRE) consensus sites existing in the promoter of human *Nrf1* gene. Two distinct transcription start sites (TSS1 and TSS2), along with translation initiation signal (TIS) are indicated by arrows in the nucleotide locations

S3 Fig

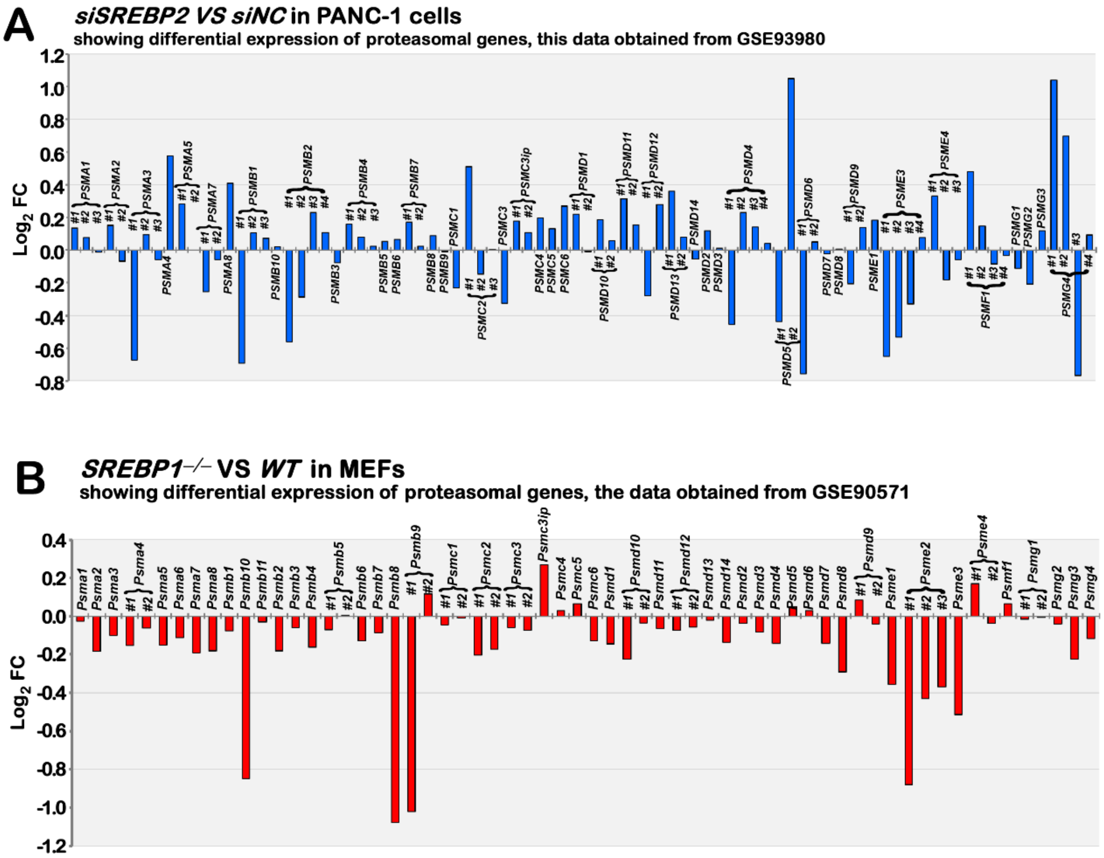

S3 Fig. Closely scrutinizing two distinct datasets reveal that almost no changes in basal transcriptional expression of all other proteasomal genes except a few of subunits were determined by transcriptomic sequencing of *siSREBP2* (A, from the GSE93980 dataset, https://www.ncbi.nlm.nih.gov/geo/query/acc. cgi? acc=GSE93980) in PANC-1 cells or *SREBP1*$^{-/-}$ MEFs (B, from GSE90571 dataset, https://www.ncbi.nlm.nih.gov/geo/query/acc.cgi?acc= GSE90571).

S4 Fig

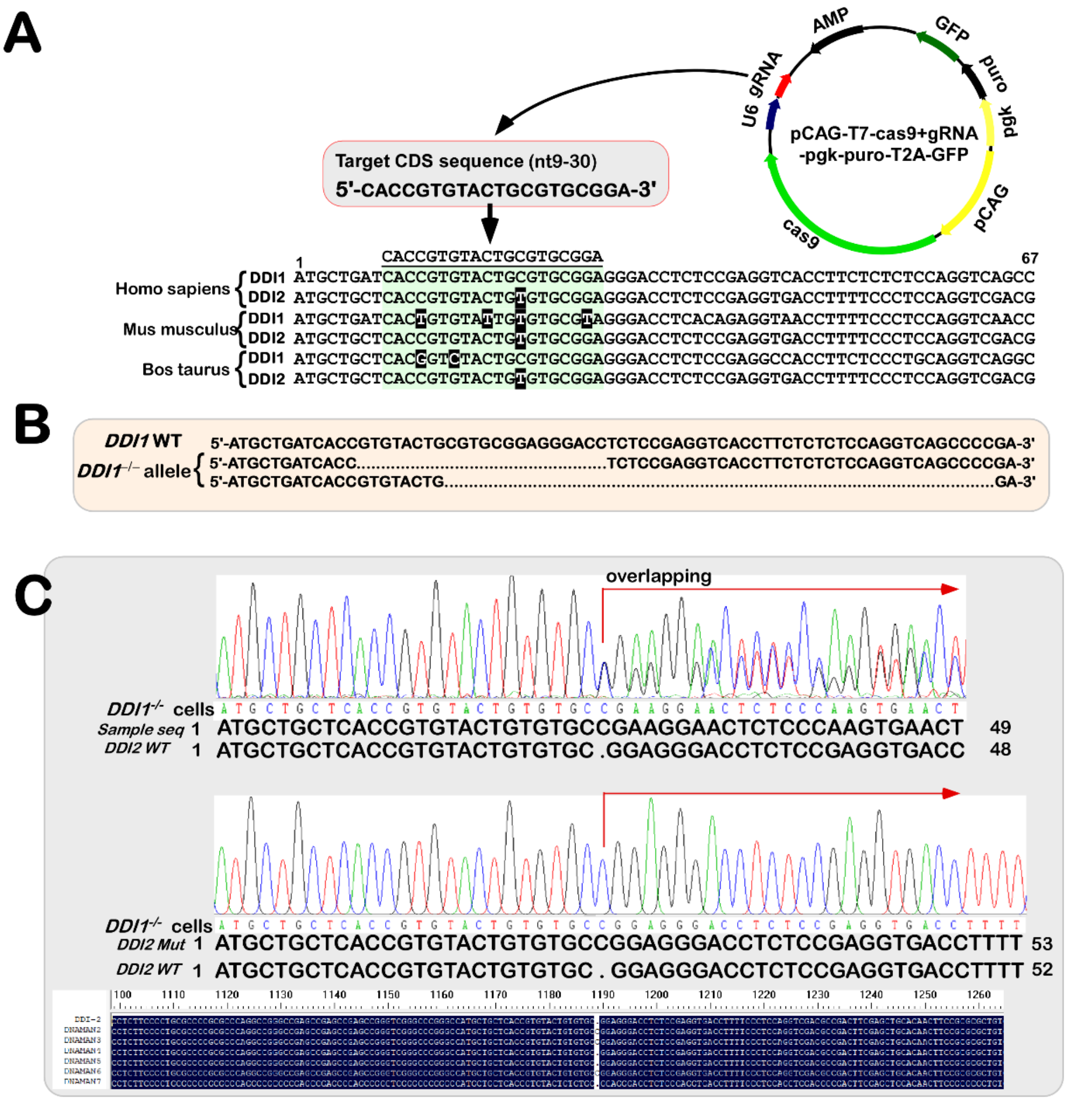

S4 Fig. The human $DDI1^{-/-}$ specific gene-editing constructs made by CRISPR/CAS9. (A) Three pairs of nucleotide sequences in CRISPR/CAS9-targeted CDS regions of *DDI1* and *DDI2* from different species are aligned. (B) The resulting knockout mutants ($DDI1^{-/-}$) were compared with its wild-type sequence.(C) An extra base of cytosine was inserted in the open-reading frame of *DDI2*, that was identified by the genomic site-specific sequencing in $DDI1^{-/-}$ cells; this was collectively designated $DDI1^{-/-}$($DDI2^{insC}$), with its location as shown by alignment of the mutant $DDI2^{insC}$ with its wildtype nucleotide sequence.

S1 Table. The key reagents and resources used in this study

Reagents or resources	Identifier	Source
**1. Oligonucleotides for qPCR**		
β -actin FW	CATGTACGTTGCTATCCAGGC	Tsingke
β -actin REV	CTCCTTAATGTCACGCACGAT	Tsingke
Nrf1 FW	TGGAACAGCAGTGGCAAGATCTCA	Tsingke
Nrf1 REV	GGCACTGTACAGGATTTCACTTGC	Tsingke
Nrf2 FW	TCAGCGACGGAAAGAGTATGA	Tsingke
Nrf2 REV	CCACTGGTTTCTGACTGGATGT	Tsingke
SREBP1 FW	ACAGTGACTTCCCTGGCCTAT	Tsingke
SREBP1 REV	GCATGGACGGGTACATCTTCAA	Tsingke
P97 FW	TGGAAACAGATCCTAGCCCTT	Tsingke
P97 REV	GCCACCAATGTCATCATACCCT	Tsingke
Hrd1 FW	ACCATCTTCATCAAGTATGTGCT	Tsingke
Hrd1 REV	TGTACACAGCCTTGTTGTCCC	Tsingke
S6K1 FW	ATCGCCACCTGTTCTTACACC	Tsingke
S6K1 REV	TCTCCCTCACCTTGCCGACCA	Tsingke
DDI-1 FW	ACCACTGTTCCCTGGGCTCCTAC	Tsingke
DDI-1 REV	ATGCTGATCACCGTGTACTGCGTGC	Tsingke
DDI-2 FW	TCCAGTGCAGTTCCCAAACTTAC	Tsingke
DDI-2 REV	ATGCTGCTCACCGTGTACTGTGTGC	Tsingke
PSMB5 FW	TCGGCAATGTCGAATCTATGAGC	Tsingke
PSMB5 REV	ATGGCTGGGGGCGCAGCGGATTGCA	Tsingke
PSMB6 FW	TCAAGAAGGAGGGCAGGTGT	Tsingke
PSMB6 REV	AGACTTCTACAACGATCCCCTCT	Tsingke
PSMB7 FW	GATACAAGAGCAACTGAAGGGATG	Tsingke
PSMB7 REV	ATGGCGGCTGTGTCGGTGTATGCTC	Tsingke
**2. Antibodies**		
Nrf1	Made by us	Zhang's Lab
Nrf2	ab62352	Abcam
p-S6K1	D151473	Sangon Biotech
β-actin	TA-09	ZSGB-BIO
P97	ab97302	Abcam
SREBP1	14088-1-AP	Proteintech
Hrd1	13473-1-AP	Proteintech
GAPDH	5174T	CST
DDI-1	H00414301-B01P	Novus Biologicals
DDI-2	A304-630A-T	Bethyl
PSMB5	ab3330	Abcam
PSMB6	ab150392	Abcam
PSMB7	ab154745	Abcam

Keap1	A1820	ABclonal
**3. Chemicals**		
Rapamycin (RAPA)	37094	Sigma Aldrich
tert-Butylhydroquinone	112941	Sigma Aldrich
MG132	M7449	Sigma Aldrich
NAC	A601127	Sangon Biotech
DMSO	67-68-5	Aladdin
**4. Cell Lines**		
HepG2	TCHu72	Cell bank of the Chinese Academy of Sciences
HL7702	GNHu 6	Cell bank of the Chinese Academy of Sciences
DDI1$^{-/-}$*(DDI2*^{insC}*)*	Made by us	In this study
**5. Oligonucleotides for siRNA**		
siSREBP1 FW	CGGAGAAGCUGCCUAUCAATT	Sangon Biotech
siSREBP1 REV	UUGAUAGGCAGCUUCUCCGTT	Sangon Biotech
Normal control FW	UUCUCCGAACGUGUCACGUdTdT	Sangon Biotech
Normal control REV	ACGUGACACGUUCGGAGAAdTdT	Sangon Biotech
**6. Oligonucleotides for expression constructs**		
Nrf1-LUC-#1 FW	CCTAGGCCTGCTAGCGCGACTGAGTTTGTCTCTACACCT	Tsingke
Nrf1-LUC-#1 REV	CTTCAGAGAAAAGCTTGCTGAAGGACCAGAATGTTTATGCT	Tsingke
Nrf2-LUC FW	CCAGGAGTTTGGTACCAGCCTGGGCAACATAGTGA	Tsingke
Nrf2-LUC REV	CCAGCTCCAAGTAGATCTTGATGAGCTGTGGA	Tsingke
SREBP1 FW	AGGAGGCGGCCGCGCCATGGACGAGCCACCCTTCA	Sangon Biotech
SREBP1 REV	GCTGAGGCCGGGGACTCTAGATCTAGCTGGAAGTGA	Sangon Biotech
**7. Recombinant DNAs**		
pcDNA3.1	V79020	Invitrogen
pGL3-Basic	VQP0121	Promega

pRL-TK	VQP0126	Promega
**8. Software and Algorithms**		
Canvas X	https://www.canvasgfx.com/	Canvas GFX, Inc.
Chromas 2.4.1	http://technelysium.com.au/wp/chromas/	Technelysium Pty Ltd.
Excel	https://www.microsoft.com/	Microsoft
Primer Premier 5	https://www.PremierBiosoft.com/	PREMIER Biosoft International
CFX Manager 3.1	https://bio-rad-cfx-manager.com/	Bio-Rad
**9. Online data**		
gRNA designed of DDI1	http://crispr.dbcls.jp/	CRISPR direct
Sequencing expression data in PANC-1 cells	https://www.ncbi.nlm.nih.gov/geo/query/acc.cgi?acc=GSE93980	GSE93980 dataset
Sequencing expression data in MEFs cells	https://www.ncbi.nlm.nih.gov/geo/query/acc.cgi?acc=GSE90571	GSE90571 dataset
**10. Others**		
Cas9/gRNA Construct Kit	VK001	v-solid
Dual-luciferase reporter assay system	E1910	Promega
RNAsimple Total RNA Kit	DP419	Tiangen Biotech
Lipofectamine®3000 Transfection Kit	L3000-015	Invitrogen
GoTaq®qPCR Master Mix	A6001	Promega
