# Supplementary material for: Nrf1 is not a direct target gene of SREBP1, albeit both are integrated into the rapamycin-responsive regulatory network in human hepatoma cells
Source: PLoS One. 2023 Nov 27;18(11):e0294508. doi: 10.1371/journal.pone.0294508 (PMC10681226; doi:10.1371/journal.pone.0294508)
Supplement: Attachment — Submitted filename: Reviewer Plo.docx [file pone.0294508.s003.docx]

7/23/2023

Editor-in Chief

PLOS ONE

Thank you very much for giving me an opportunity to review **Manuscript Number:**  PONE-D-23-03948 , entitled:” Nrf1 is not a direct target gene of SREBP1, albeit both are integrated into the rapamycin-responsive regulatory network in human hepatoma cells.”

In this manuscript, the authors reported that Nrf1is not a direct target of SREBP1.

Other points are:

Abstract section:

It is suggested that abstract be written more clearly .

Introduction section:

The introduction needs to review more articles.

Also, sentence “Nrf1 is not a direct target of SREBP1, albeit they are involved in the rapamycin-responsive regulatory networks “should be clarified.

Methods section:

It is suggested to write a reference for each method.

Results and Discussion section:

How did the authors conclude that SREBP1 may be directionally responsible for regulating protein synthesis and degradation?

This sentence “Nrf1 is not a direct target of SREBP1, albeit its indirect effects on upstream signaling to Nrf1 cannot be ruled out” needs more explain.

It would be better if revise the conclusion section.

This MS needs revision.

With bests wishes

Dr.Durdi Qujeq

Faculty of Medicine , Babol University of Medical Sciences
